# Friction Stir Joining of Structural Polymers and Aluminum Alloys—A Direct Comparison of Mechanical Behavior and Rheological Effects on Dissimilar Metal–Polymer Joints

**DOI:** 10.3390/polym18161993

**Published:** 2026-08-16

**Authors:** Arménio N. Correia, Bárbara Coelho, Catarina R. Leal, Susete N. Fernandes, Virgínia Infante, Pedro Vilaça

**Affiliations:** 1 LAETA, IDMEC, Instituto Superior Técnico, Universidade de Lisboa, 1049-001 Lisboa, Portugal; 2 Instituto Superior Técnico, Universidade de Lisboa, 1049-001 Lisboa, Portugal; 3 Departamento de Física, Instituto Superior de Engenharia de Lisboa, Instituto Politécnico de Lisboa, 1959-007 Lisboa, Portugal; 4 Centro de Física e Engenharia de Materiais Avançados, Instituto Superior Técnico, Universidade de Lisboa, 1049-001 Lisboa, Portugal; 5 CENIMAT/I3N, NOVA School of Science and Technology, NOVA University Lisbon, 2829-516 Caparica, Portugal; 6 Department of Materials Science, NOVA School of Science and Technology, 2829-516 Caparica, Portugal; 7 Department of Energy and Mechanical Engineering, School of Engineering, Aalto University, 02150 Espoo, Finland

**Keywords:** friction stir joining, hybrid joints, rheology, AA6082-T6, PEEK, Noryl GFN2

## Abstract

The continuous joining of aluminum alloys to engineering thermoplastics has emerged as a promising manufacturing path for lightweight hybrid structures, yet the influence of polymers’ mechanical behavior on friction stir joining remains poorly understood. This work investigates the role of melt rheology on the morphology, joining interface, and mechanical strength of dissimilar joints that combine AA6082-T6 with two engineering-grade thermoplastics, Noryl^®^ GFN2 and SustaPEEK^®^. Two joining strategies were assessed under identical processing conditions: conventional friction stir joining (FSJ) and through-slot friction stir joining (TS-FSJ), the latter incorporating thin titanium strips intended to reduce heat transfer to the polymer. Joint morphology was assessed by optical and scanning electron microscopy, mechanical performance was evaluated through quasi-static tensile-shear testing, and the rheological behavior of both polymers was characterized by steady shear and oscillatory measurements. Conventional FSJ produced defect-free aluminum–Noryl joints, with a mechanical strength of 111.3 ± 8.4 kN/m, whereas aluminum–PEEK joints exhibited localized polymer overflow, poor surface finish and scattered strength performance of 116.7 ± 77.2 kN/m. Rheological measurements showed that PEEK exhibited higher melt viscosity and viscoelastic moduli, restricting polymer flow and promoting unstable interface formation. Although titanium inserts reduced heat transfer in TS-FSJ, their deformation reduced the effective joining area, resulting in lower tensile strength. Polymer rheology was identified as one of the key factors governing material flow, defect formation, process stability, and the joints’ mechanical performance, emphasizing the importance of tailoring the processing parameters reflecting the rheological characteristics of each polymeric base material.

## 1. Introduction

The demand for lightweight, multifunctional structures in the transportation, aerospace, and electrical industries has accelerated the development of hybrid metal–polymer systems, where aluminum alloys are combined with high-performance thermoplastics so that improved specific strength, corrosion resistance, and functional integration can be attained [1,2]. Particularly, the aluminum alloy AA6082-T6 is widely used in structural applications due to its good strength-to-weight ratio alongside its notable corrosion resistance. Engineering polymers such as polyether ether ketone, PEEK, and polyphenylene ether-based blends, like the Noryl^®^ series, offer low density and chemical stability with excellent design flexibility [3,4]. However, the underlying mismatch in thermal, mechanical, and chemical properties between metals and polymers represents a major limitation of conventional joining technologies [5,6].

Traditional joining techniques, including mechanical fastening and adhesive bonding, are widely applied but present important drawbacks. Mechanical fastening introduces stress concentrations, requires drilling operations, and increases structural weight, while adhesive bonding requires extensive surface preparation, curing time, and often suffers from long-term degradation under hot and humid environments. Fusion welding is not applicable due to the large difference between the melting temperature of aluminum alloys and the degradation temperature of thermoplastics, which leads to polymer decomposition before metallic melting can occur [7,8,9].

Solid-state joining technologies have therefore emerged as promising alternatives, with friction-stir-based technologies, such as friction stir welding (FSW) or friction stir spot joining (FSSJ) gaining traction, not only within the academic domain but also in high-end technological sectors such as the aerospace sector [10,11,12]. These joining processes rely on frictional heating and severe plastic deformation to soften both the polymeric and metallic base materials to enable the fundamental joining mechanism between them. This is governed by mechanical interlocking, where the softened polymer flows into surface asperities generated on the metallic counterpart, being clinched after re-solidification upon cooling [13,14].

The quality of friction-stir-based joints strongly depends on processing parameters such as rotational speed, travel speed, tool geometry, or plunge depth, all contributing to the required heat generation and material flow. Excessive heat input may result in polymer thermal degradation, void formation, and loss of interfacial confinement, whereas insufficient heat prevents adequate polymer softening and its ability to be extruded into villi-shaped features on the metallic surface. These thermal limitations are particularly critical in polymer-based systems due to the narrow processing window between adequate softening and degradation [4,5].

Contrary to metallic friction stirred joints, where the thermo-mechanical behavior has been extensively studied in metallic systems [15], in metal–polymer joints, the presence of a viscoelastic material introduces additional complexity due to temperature-dependent rheological behavior, including viscosity reduction, shear thinning, and melt flow instability [16,17], making polymer flow a key factor in interface formation and defect evolution.

Among high-performance thermoplastics, PEEK has been extensively studied due to its high melting temperature, excellent mechanical performance, and thermal stability. The aluminum–PEEK joints produced by friction-stir-based processes have demonstrated that mechanical interlocking is the dominant binding mechanism, with polymer flow filling the surface cavities generated on the aluminum substrate [18,19]. Nonetheless, excessive heat input may lead to polymer degradation and gas formation, with the development of voids with reduced mechanical strength [3].

Correia et al. [14] contributed to the understanding of aluminum–polymer friction stir joining mechanisms, demonstrating how tool penetration depth and rotational speed strongly influence joint morphology and mechanical performance. It was also highlighted that improper control of process parameters leads to polymer overflow through the joining line, excessive flash formation, and reduced effective binding area. These findings underline the importance of maintaining a stable thermal window during processing, confirming, on the one hand, that excessive heat input can generate an oversized molten polymer pool, reducing the confinement conditions that induce the loss of boundary conditions among the base materials, which negatively affects the resulting joint integrity [3]. On the other hand, too low heat input leads to poor material flow and hindering of the ability to induce micromechanical interlocking among base materials [20], indicating that the rheological behavior of the polymeric-based materials may significantly affect the development of joining mechanisms. Moreover, such kinds of joints also demonstrated that mechanical performance is highly sensitive to strain rate and loading conditions [21,22], confirming that polymer flow behavior plays a critical role in determining joint strength and failure mode.

From a broader perspective, rheology can be considered a key factor controlling material behavior during friction-stir-based joining processes [23,24,25]. Particularly in the case of polymeric-based materials, viscosity, shear thinning response, and molecular mobility govern the ability of the polymer to flow into metallic asperities under the applied forging pressure. When viscosity is too high, insufficient filling occurs, whereas overly low viscosity or excessive heating may cause uncontrolled material overflow and void formation [26,27].

Despite the significant progress achieved in combining metals and polymers using friction-stir-based technologies in recent years, there is still a lack of comparative studies addressing the influence of the rheological behavior of different polymeric materials under identical processing conditions. Particularly, the combined effects of thermal input, weld pool development, and polymer rheology on interfacial stability and mechanical performance remain insufficiently understood when combining such base materials with aluminum alloys. This knowledge gap limits the understanding of the underlying mechanisms governing the material flow and development of interfacial binding mechanisms and, consequently, the resulting mechanical performance. As a result, the underlying assumption is that differences in viscosity- and temperature-dependent flow behavior may significantly affect the ability of the polymer to conform to and fill the surface features of the aluminum surface, thereby influencing joint morphology and mechanical performance. This provides the basis for comparing different polymers under the same processing conditions and assessing the role of rheological behavior in their joinability.

The scope of this work was to investigate overlapped friction stir joints combining AA6082-T6 to SustaPEEK^®^ and AA6082-T6 to Noryl^®^ GFN2, aiming to (i) compare interfacial formation mechanisms between semicrystalline and glass-fiber-reinforced polymers, (ii) evaluate the effect of using titanium strips to shield the polymeric material, thus controlling the weld pool size and boundary confinement, and (iii) correlate polymer rheological behavior with joint morphology and mechanical performance. The outcomes provide insights into the role of thermal management and polymer flow behavior during friction stir joining of metal–polymer joints.

## 2. Materials and Methods

### 2.1. Thermoplastic Systems

Two structural thermoplastic materials: (i) Noryl^®^ GFN2 (PHT-Plastiques Hautes Technologies, France—Noryl from here on) and (ii) SustaPEEK^®^ (Roechling Industrial, Italy—PEEK from here on), both with 5 mm (−0.0, +0.2 mm) nominal thickness, were combined with a medium-strength aluminum alloy, AA6082-T6 (Poly Lanema, Portugal), with 2 mm (−0.0, +0.05 mm) nominal thickness. Noryl GFN2 is a polymeric composite material blending poly(phenylene-ether) (PPE) and high-impact polystyrene (PS), randomly reinforced with 20% weight of short glass fiber, resulting in an amorphous thermoplastic with uniform properties. Given the proprietary nature of Noryl, the exact blend is unknown [28,29]. The glass fiber reinforcement combined with the amorphous nature of thermoplastic matrix of Noryl significantly influences melt flow behavior and interfacial consolidation, evidencing complex rheological behavior due to fiber orientation, heterogeneous viscosity, and localized flow restriction [30]. PEEK (poly(ether-ether-ketone)) is a semi-crystalline thermoplastic belonging to the polyaryletherketone (PAEK) family. Its aromatic molecular structure and semi-crystalline morphology provide high thermal and mechanical stability, while also distinguishing its processing behavior from that of amorphous polymers such as Noryl. These differences are particularly relevant when considering the rheological response and joint formation during solid-state joining [19,31].

### 2.2. Production Set-Up

**Figure 1:**
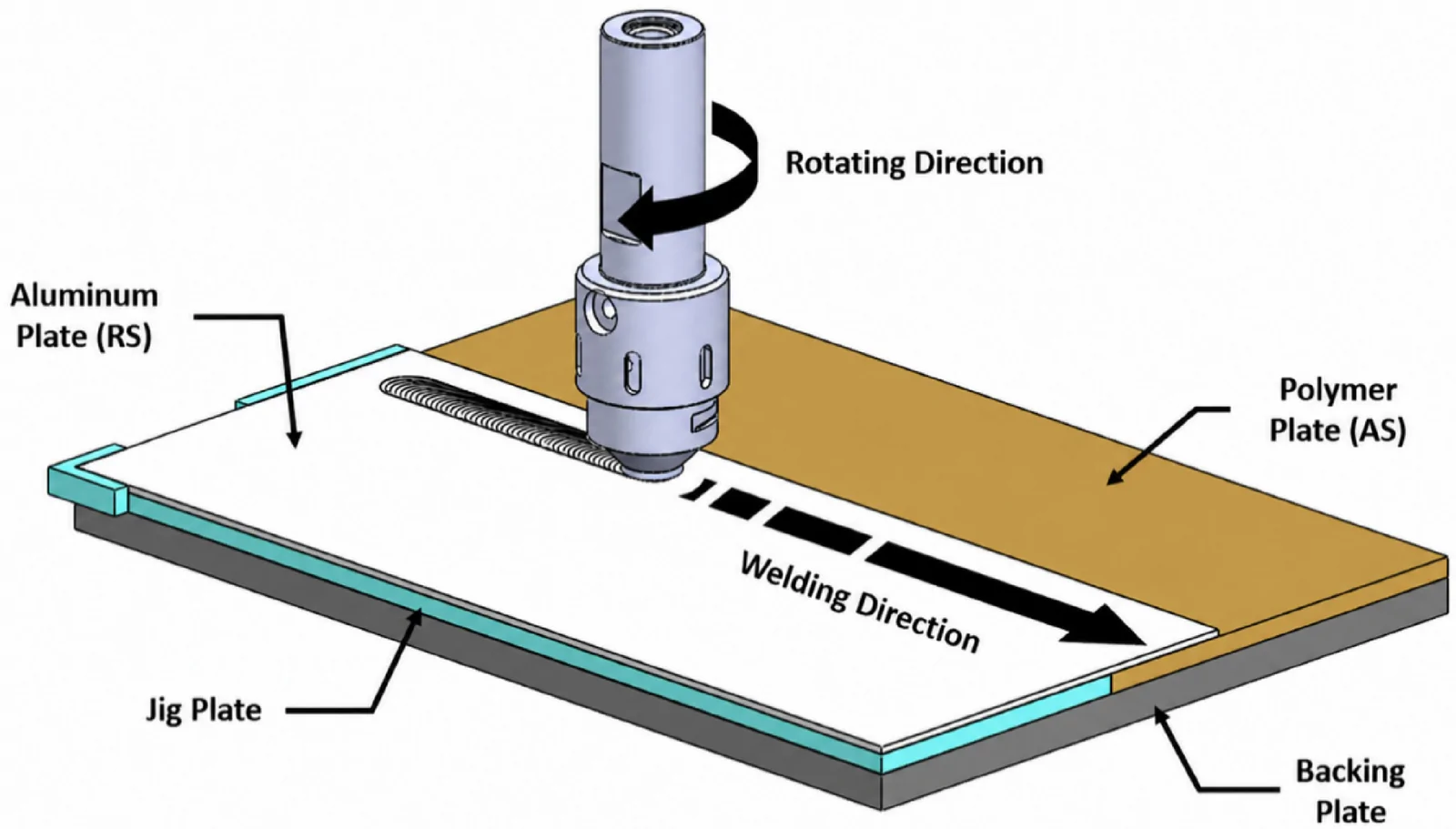
Schematic of overlapped friction stir joining setup—not to scale.

The fabrication process employed an ESAB^®^ Legio FSW 5U (MD, USA) machine equipped with a modular tool composed of a 1.5 mm long and 5 mm diameter threaded cylindrical pin, attached to a flat scrolled shoulder 24 mm in diameter. The metallic and polymeric sheets were cut into 200 mm × 125 mm plates and, based on previous research, were joined in overlap configuration, placing the metallic plate (advancing side—AS) over the polymeric plate (retreating side—RS) [3,5]. The joining process was position-controlled using the set-up schemes in Figure 1, employing the set of parameters listed in Table 1.

**Figure 2:**
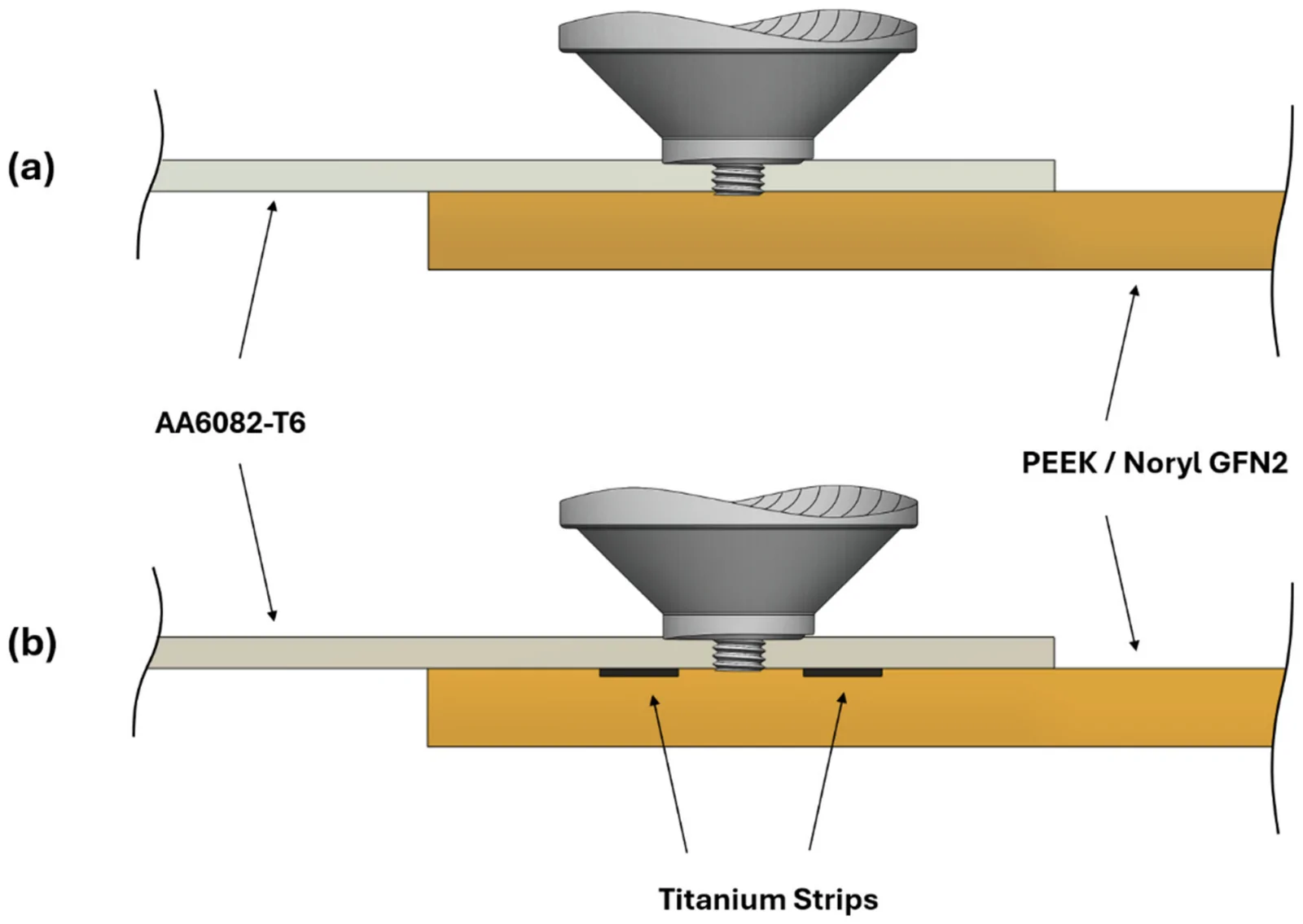
Joining strategies’ cross-sectional view—(**a**) conventional overlapped friction stir joining (FSJ) and (**b**) through-slot friction stir joining (TS-FSJ)—not to scale.

Two joining strategies were adopted: (i) conventional overlapped friction stir joining, and (ii) through-slot friction stir joining. The latter is based on Khadka et al. [3], which incorporates two titanium strips 0.6 mm thick inserted into milled channels in the interfacial face of the polymeric base material along the joining line. These strips act as a thermal shield of the polymeric base materials to avoid excessively large molten pools that may induce the loss of boundary conditions among base materials. A schematic of the production setup and joints’ configuration is displayed in Figure 2, whereas the main physical and thermo-mechanical properties of the base materials are summarized in Table 2.

### 2.3. Tensile Testing

**Figure 3:**
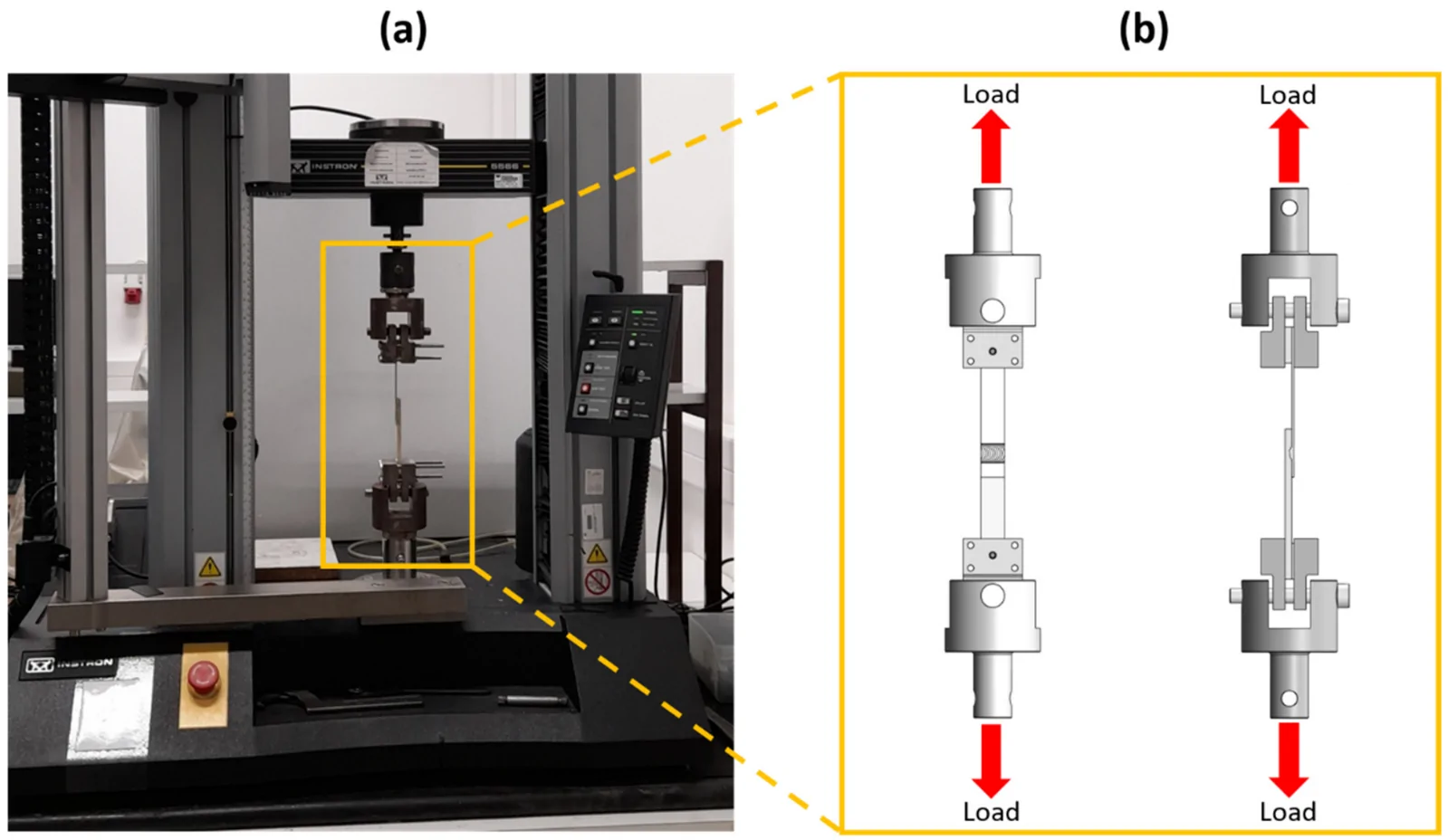
(**a**) Quasi-static tensile testing setup and (**b**) detailed schematic—not to scale.

After joining, the composite panels were cut perpendicular to the travel direction into 20 and 5 mm-wide specimens. The 20 mm-wide specimens were subjected to quasi-static tensile tests at 5 mm/min using an Instron^®^ 5566 universal testing machine (Norwood, MA, USA) equipped with a 10 kN load cell, as detailed in Figure 3, while the 5 mm ones were cold-mounted, polished and submitted to scanning electron microscopy (SEM) using an analytical SEM Hitachi^®^ S2400 (Tokyo, Japan).

### 2.4. Rheological Testing

The rheological characterization of both Noryl and PEEK in the melt state was performed with a Modular Compact Rheometer MCR 502 (Anton Paar, Madrid, Spain), equipped with an electric plate and a thermal chamber for temperature control. A parallel-plate geometry, with a diameter of 25 mm and a gap of 2.0 mm, was used. Oscillatory shear measurements were performed to evaluate the storage (G′) and the loss (G″) moduli within the linear viscoelastic regime to ensure moduli and strain independency. Angular frequency sweep measurements were carried out under a constant strain of 0.5% with frequency ranging from 0.001 and 30 Hz. Shear flow measurements were performed to characterize the viscosity curve in the shear rate range of 0.01 to 100 s^−1^. Previously, a pre-shear of 10 s^−1^ during 60 s followed by an equilibration of 120 s. based on previous studies reporting peak processing temperatures of up to 309.9 °C on the advancing side [5,20]. Considering that the temperature at the joint center was expected to be even higher, the measurements were carried out at 350 °C, and, after sample loading, the equilibration time was respected to guarantee temperature equilibration. Given the inherent difficulty of measuring the interfacial processing temperature, the testing temperature was selected based on previous research in which the processing temperature was thoroughly assessed over a wide range of processing parameters during friction stir joining between metals and thermoplastic polymers [5,20].

## 3. Results and Discussion

### 3.1. Joint Morphology

**Figure 4:**
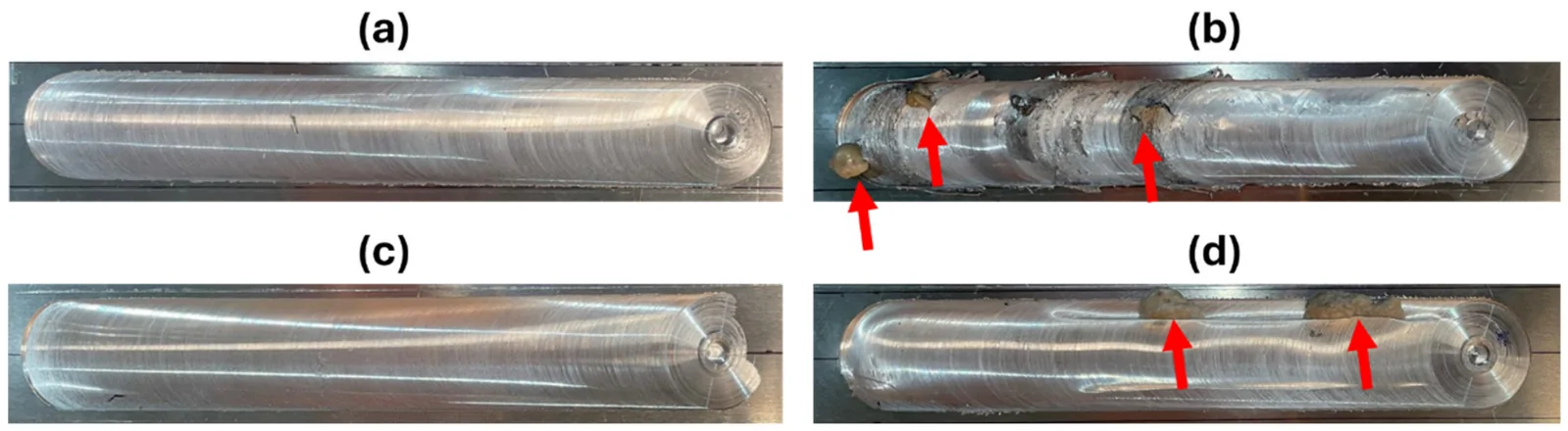
Surface quality of seams produced by conventional friction stir joining and through-slot friction stir joining. Conventional friction stir joining: (**a**) aluminum-Noryl and (**b**) aluminum-PEEK. Through-slot friction stir joining with titanium strips: (**c**) aluminum-Noryl and (**d**) aluminum-PEEK.

Figure 4 depicts the surface quality of aluminum–polymer joints produced either by conventional friction stir joining or through-slot friction stir joining. The aluminum–Noryl joints depicted in Figure 4a,c exhibit smooth and continuous joining surface with no apparent surface defects or polymeric overflow, indicating stable material flow throughout the process. In contrast, aluminum–PEEK displayed in Figure 4b,d shows several spots of polymeric overflow, indicated with red arrows.

Since the process was position-controlled, these overflow spots may indicate a temperature build-up beneath the shoulder that decreased the amount of forging force required to maintain the vertical position of the tool. As a result, it allowed the softened polymer to flow outward, resulting in the observed spots of polymer overflow in the conventional FSJ of aluminum and PEEK, in line with previous observations of Correia et al. [14]. The incorporation of titanium strips in the TS-FSJ configuration modified the material flow by shielding the heat flow towards the polymeric base material, constraining the lateral flow around the joining line, which seems to have helped stabilize the stirring process, resulting in improved surface quality. Nonetheless, polymeric overflow was still observed in the TS-FSJ of PEEK, particularly at the edge of the titanium strip placed on the AS of the joint. Since the tool stirring was maximum on this side [10], temperature build-up and decrease in required forging force were also expected, which may explain this behavior.

**Figure 5:**
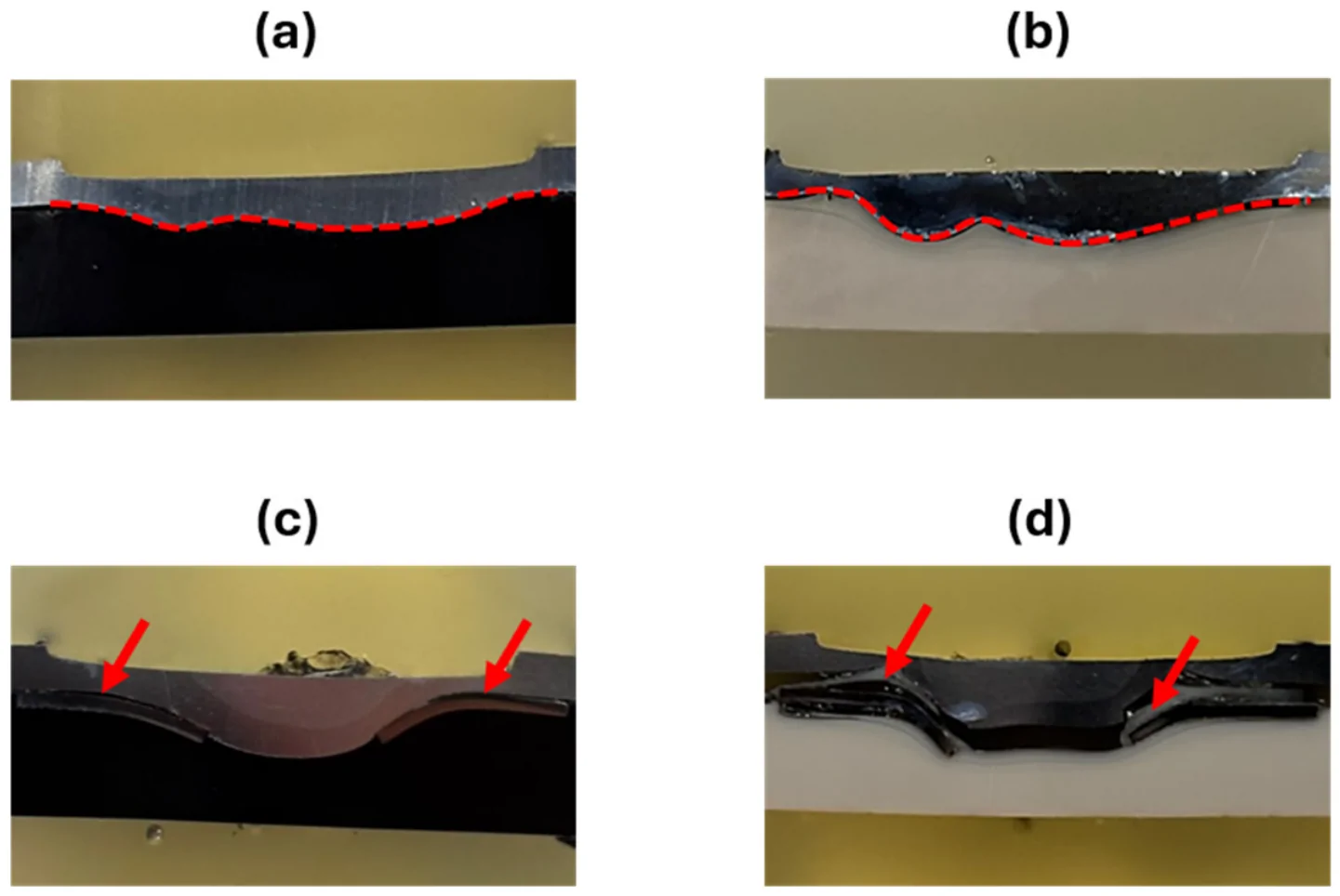
Cross-sectional morphology of joints produced by conventional friction stir joining and through-slot friction stir joining. Conventional friction stir joining: (**a**) aluminum–Noryl and (**b**) aluminum–PEEK. Through-slot friction stir joining with titanium strips: (**c**) aluminum–Noryl and (**d**) aluminum–PEEK.

In the cross-sectional morphology of the joints produced, a double concavity-shaped interface was clearly visible among the base material in conventional friction-stirred joints, indicated with dashed red lines in Figure 5a,b, which is a characteristic morphology previously reported in early research by Correia et al. [5,32].

Observation of the TS-FSJ joints revealed that the titanium strips were plastically bent during the joining process due to the vertical forging force exerted by the shoulder, which allowed softened polymer to flow around it, as indicated with red arrows in Figure 5c,d. As a result, the titanium strips became embedded in the polymeric base materials, preventing effective joining with the aluminum plate positioned above them and consequently reducing the effective joining area.

### 3.2. Quasi-Static Tensile Tests

**Figure 6:**
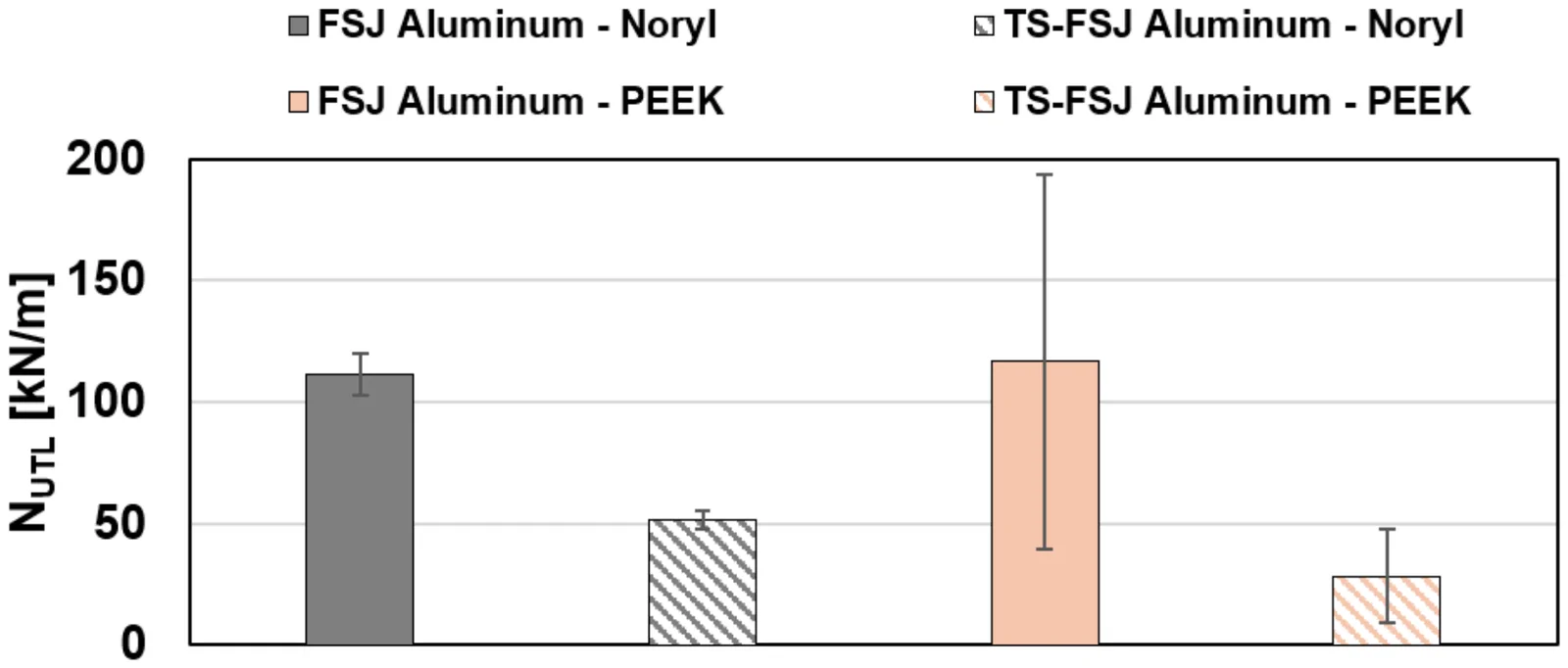
Length-normalized ultimate tensile load (NUTL) for each joining condition and base material pair.

The quasi-static tensile tests revealed that the conventional FSJ of aluminum and Noryl had an average length-normalized strength of 111.3 kN/m, while the combination of aluminum with PEEK reached a slightly higher strength of 116.7 kN/m. Moreover, the mechanical performance of aluminum–Noryl joints was found to be very homogeneous, with a standard deviation of 8.4 kN/m. Conversely, the aluminum–PEEK joints evidenced scattered performance, resulting in a significant standard deviation of 77.2 kN/m, as observable in Figure 6.

**Figure 7:**
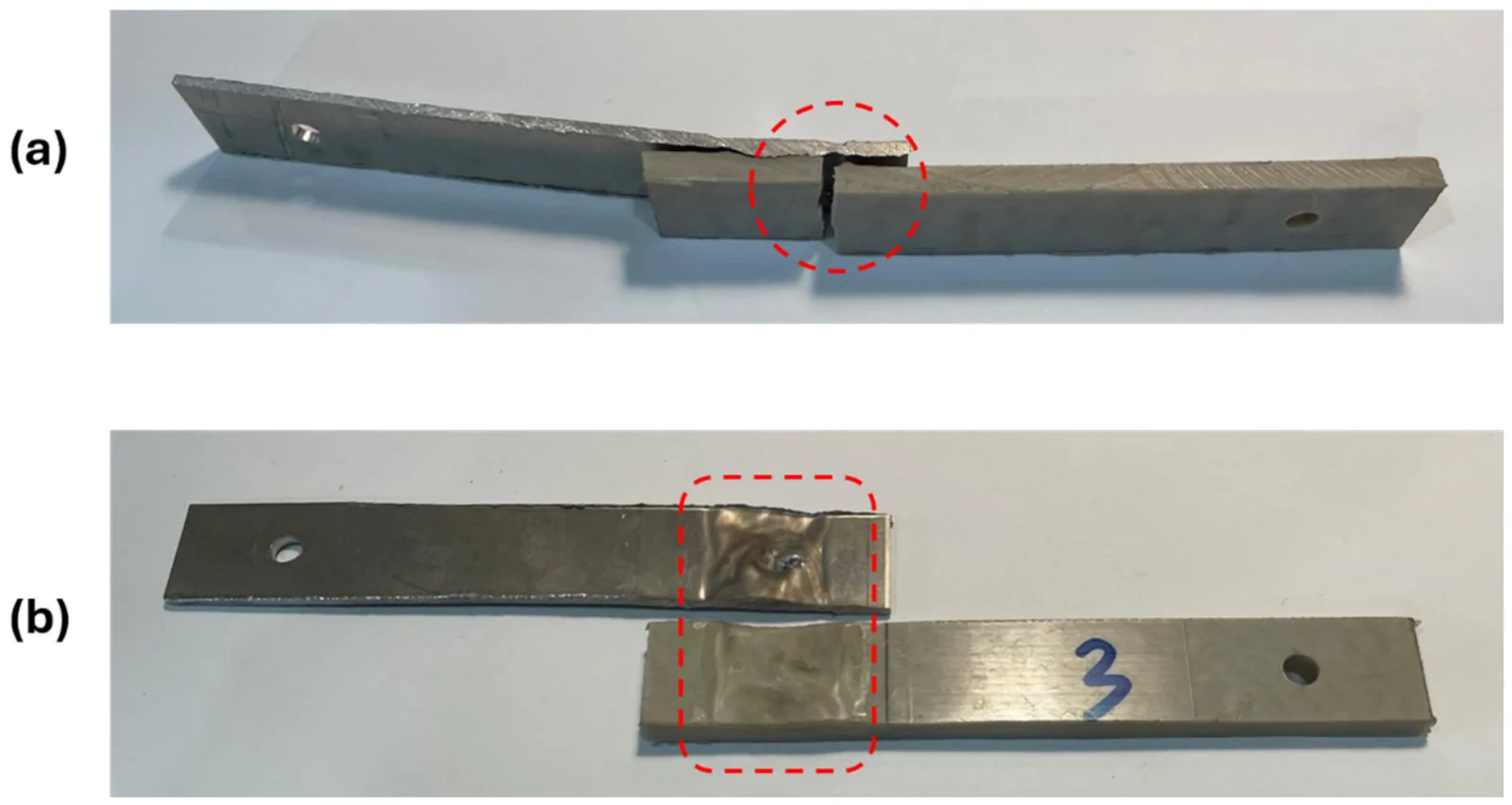
(**a**) A sample of an aluminum–PEEK joint produced using FSJ with fracture on the polymeric end close to the joining region, and (**b**) a sample of an aluminum–PEEK joint produced using FSJ with interfacial failure.

These results appear to be highly influenced by both the surface quality and morphology of the joints discussed in Section 3.1, since the failure mechanisms observed in high-strength joints were found to be notably different from those with lower strength. All aluminum–Noryl joints produced by FSJ evidenced the same failure mechanism, characterized by a crack nucleating in the transition region between the joint and the low-stiffness region associated with the polymeric plate. The crack nucleation resulted from the increased local stress within this region due to the combination of both tensile and bending stresses, the latter one induced by the secondary bending moment. This secondary bending moment is induced by geometrical asymmetry typical of overlap joints and stiffness asymmetry among metallic and polymeric base materials [33,34]. For the aluminum–PEEK joints, also produced by FSJ, it was observed that those with higher mechanical strength evidenced the same failure mechanism identified above, yet the ones with lower mechanical performance displayed a failure mechanism in which the joining area was detached throughout its interface. This failure mode occurs when the binding strength among the metallic and polymeric base materials is unable to withstand the loads imposed by the tensile test. A further analysis of the aluminum–PEEK joints with interfacial failure evidenced that the joining interface was very irregular, suggesting an unstable stirring process, in line with the surface quality shown in Figure 4b and the results displayed in Figure 6. The observed failure mechanisms are depicted in Figure 7.

**Figure 8:**
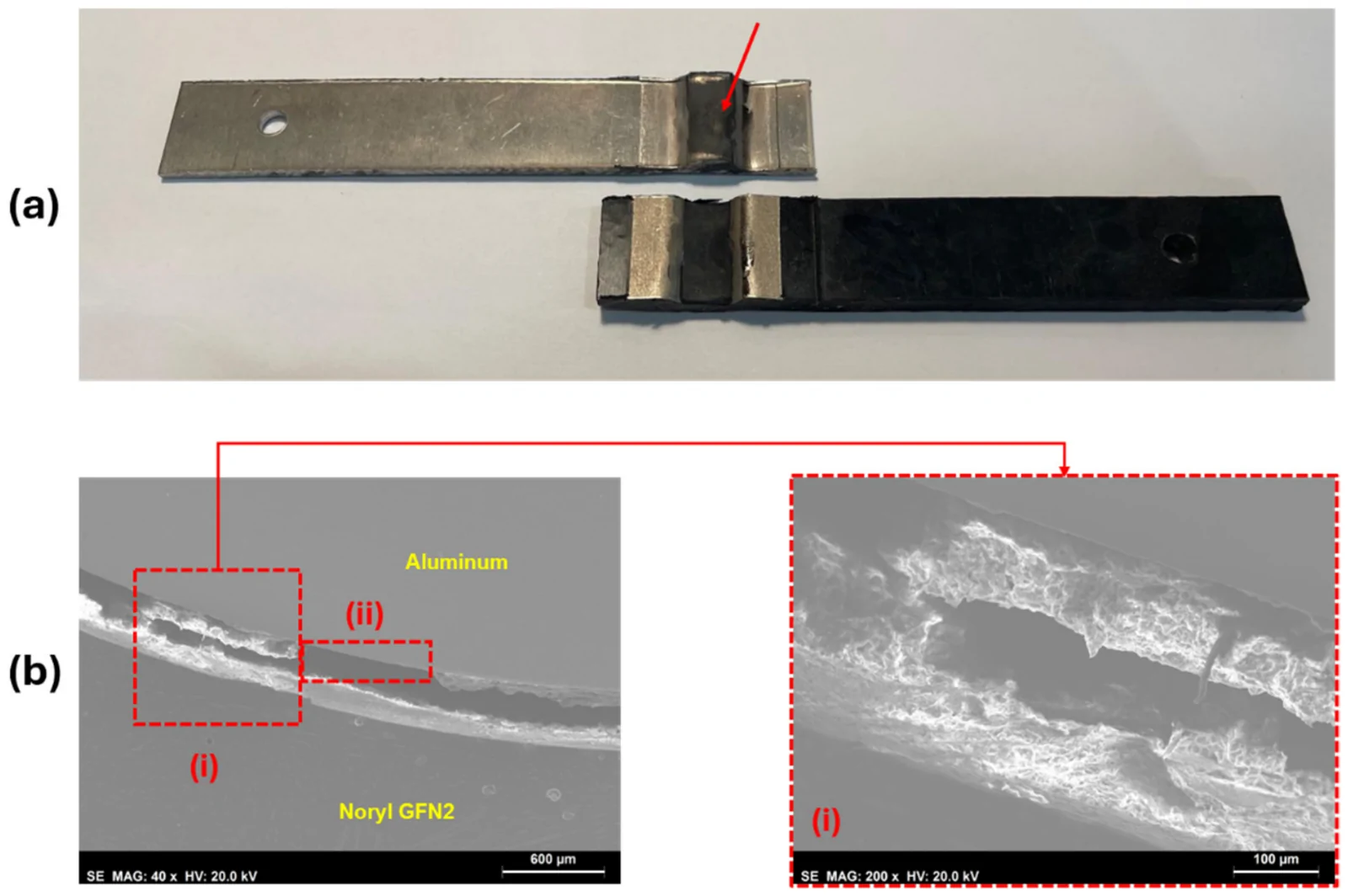
(**a**) Sample of aluminum–Noryl TS-FSJ joint with interfacial failure and (**b**) SEM images of interfacial failure.

For the TS-FSJ joints, the quasi-static tensile tests evidenced a significant drop in average strength compared to the counterpart joints produced via conventional FSJ. The aluminum–Noryl joints with titanium strips reached and average mechanical strength of 51.5 kN/m, while the aluminum–PEEK joined in the same configuration evidenced a value of 28.3 kN/m. This significant drop in mechanical performance resulted from the reduced effective joining area observed in Figure 5c,d. As a result, the amount of micro-interlocking features developed could not withstand both the shear and peeling stresses developed, leading to interfacial failure across the base materials, as presented above. Figure 8 depicts an example of interfacial failure observed on an aluminum–Noryl joint produced by TS-FSJ, as well as scanning electron microscopy images of the interface region.

A visible layer of Noryl (110 μm thickness) adhered to aluminum was observed and is depicted in Figure 8a, indicated with a red arrow. Nonetheless, after SEM analysis, it was found that the regions where the polymeric base material adhered to the aluminum surface was not continuous, as highlighted in Figure 8b in the dashed red boxes (i) and (ii). This observation supports the drop in mechanical strength of the joints produced by TS-FSJ, regardless of the base material pairs, while suggesting some degree of flowing capacity of the polymeric materials that influences the ability to develop binding mechanisms with the aluminum plates.

Table 3 shows that the normalized ultimate tensile load (N_UTL_) obtained by conventional FSJ in the present research is comparable to the results reported in the literature for both aluminum–Noryl and aluminum–PEEK joints. The strength results fall within the range reported for comparable dissimilar material joints, ranging from 100 to 137 kN/m, although they are around 14 to 18% lower than the highest values reported. Nevertheless, it should be noted that in this study suboptimal processing parameters were used, which reinforces the obtained results, demonstrating that the proposed joining conditions provide tensile performance comparable to state-of-the-art conventional FSJ.

Conversely, the TS-FSJ joints exhibited notably lower tensile strength compared not only to the conventional FSJ counterparts but also to the 110 kN/m reported by Khadka et al. [3] when joining AA5754-H111 and PEEK by TS-FSJ. The observed under-performance of joints produced by TS-FSJ might be related to the fact that, in the present study, the aluminum plate was only 2 mm thick, not offering enough stiffness during the stirring process, resulting in the bending of the titanium strips. The bent strips, in turn, allowed the polymeric material to flow around them, forming a thin layer of polymer that hindered the mechanical performance of the joints.

### 3.3. Rheological Characterization

**Figure 9:**
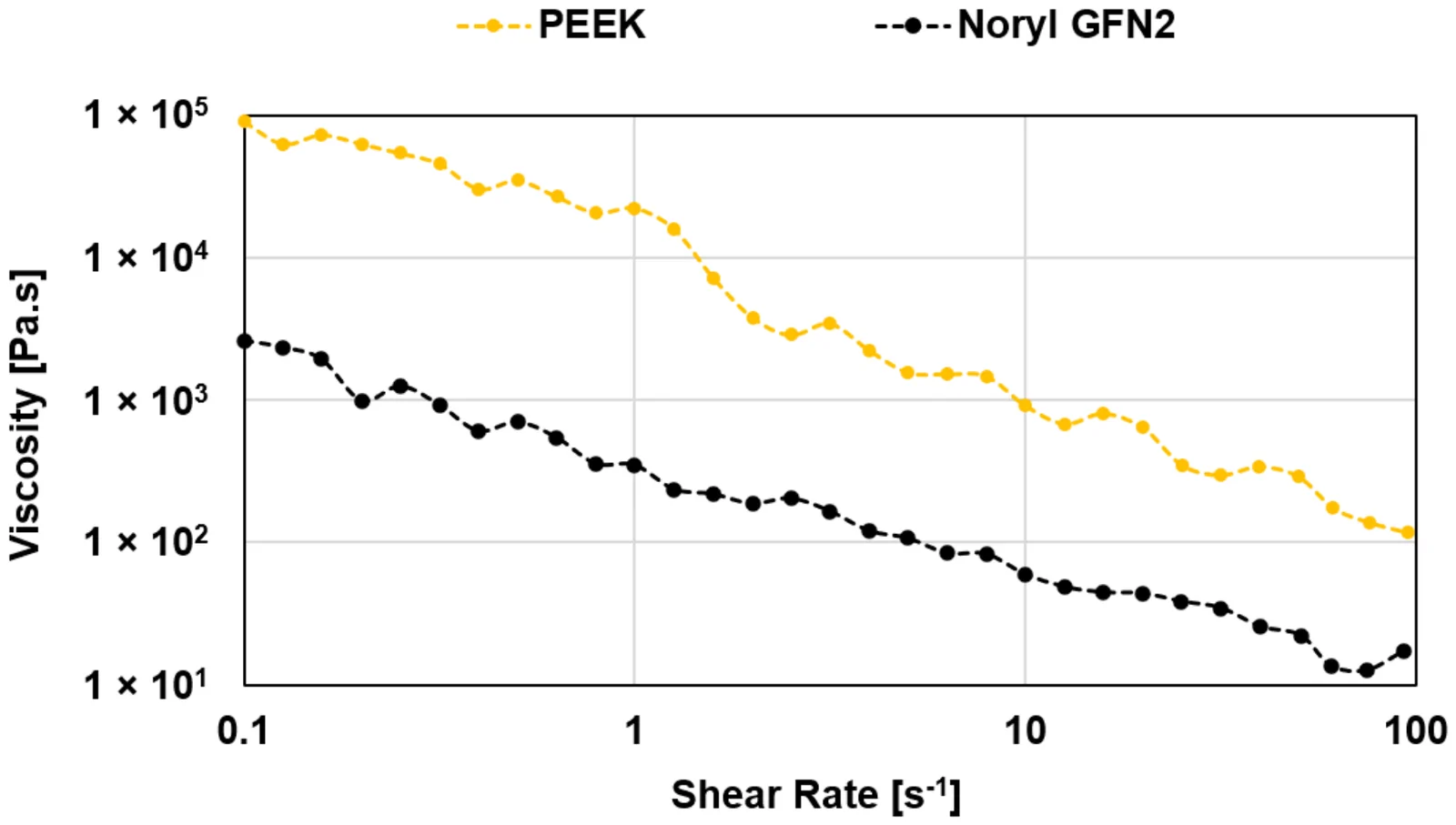
Steady shear flow behavior: viscosity as function of shear rate of Noryl GFN2 and PEEK, at 350 °C.

To better understand the obtained results so far, a set of steady and dynamic rheological tests were carried out, both on Noryl GFN2 and PEEK polymeric base materials. Figure 9 displays the steady shear viscosity as a function of shear rate, with both base materials exhibiting pronounced non-Newtonian shear-thinning behavior, characterized by a continuous decrease in viscosity with increasing shear rate. This behavior is typical of polymer melts, where increased shear promotes molecular chain orientation and disentanglement, reducing its resistance to flow [22,30].

Although PEEK is not fiber-reinforced, it demonstrates substantially higher viscosity than Noryl GFN2, which is fiber-reinforced, across the entire investigated range of shear rate. On the one hand, the consistently higher viscosity of PEEK reflects its greater molecular rigidity and stronger intermolecular interactions, which result in enhanced resistance to deformation under shear. On the other hand, the lower viscosity of Noryl GFN2 enables improved material characteristics under comparable thermo-mechanical conditions.

**Figure 10:**
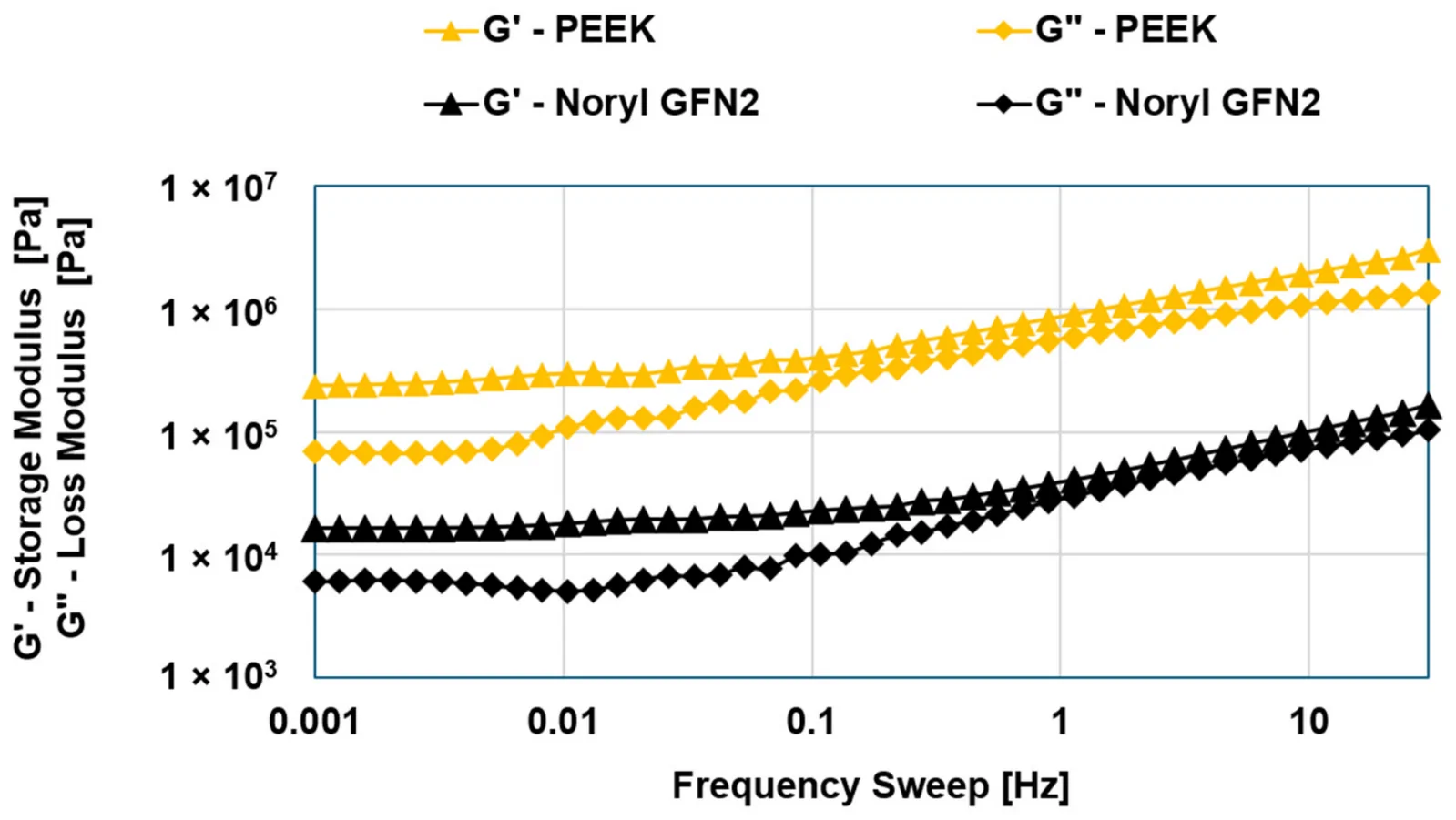
Oscillatory flow behavior: frequency sweep tests—storage modulus (G′) and loss modulus (G″) as function of frequency of Noryl GFN2 and PEEK at 350 °C.

When analyzing the dynamic behavior of these polymeric base materials, namely, the storage modulus (G′) and loss modulus (G″), it was found that both parameters increase with increasing oscillatory frequency. This behavior, depicted in Figure 10, reflects the viscoelastic response of polymer melts and can be explained by the reduced relaxation time available for molecular chain rearrangement at higher deformation rates.

PEEK demonstrates significantly higher storage and loss moduli compared to Noryl GFN2 over the entire frequency range. For both materials, the storage modulus remains consistently higher than the loss modulus, indicating that the elastic component of the viscoelastic response dominates over the viscous component. The substantially higher moduli observed for PEEK indicate greater viscoelastic stiffness with improved resistance to deformation, whereas Noryl GFN2 exhibits a more compliant viscoelastic response.

These rheological characteristics are consistent with the higher melt viscosity of PEEK observed in the steady shear measurements and further confirm its superior mechanical rigidity relative to Noryl GFN2 and seem to correlate with the joints’ morphology and tensile performance observed. Given that PEEK exhibits significantly higher viscosity and viscoelastic moduli than Noryl GFN2, conventional FSJ of aluminum–PEEK produced more surface defects and less-uniform material flow than that of aluminum–Noryl joints (Figure 4). These morphological differences are signaled in the tensile results displayed in Figure 6, where aluminum–Noryl joints exhibit similar average normalized ultimate tensile loads compared to aluminum–PEEK joints. Since Noryl GFN2 has 20% less ultimate tensile strength compared to PEEK and all joints were produced with the same processing parameters, it can be stated that the rheological behavior of both polymeric materials played a critical role in the observed results. A comparison of both joining processes is summarized in Table 4.

## 4. Conclusions

The present research was designed to address the effects of the rheological behavior of polymeric base materials when joined with aluminum ones employing friction-stir-based technologies. Two joining strategies were assessed for both pairs of base materials—(i) conventional FSJ and (ii) TS-FSJ—using the same set of processing parameters.

It was observed that the polymers’ rheology plays a key role in governing friction stir joining performance in dissimilar metal–polymer joints. Compared to Noryl GFN2, PEEK exhibited higher melt viscosity and viscoelastic moduli, which limited its ability to flow under a thermo-mechanical joining process, promoting the development of localized defects such as polymeric overflow. Although PEEK has higher tensile strength than Noryl, the aluminum–PEEK joints did not consistently outperform the aluminum–Noryl ones due to the lower flow characteristics of PEEK that generated defects that reduced structural integrity, leading to volatile tensile results.

The titanium strips acted as thermal shields but also reduced the effective binding area after stirring, resulting in lower tensile strength when compared with the counterpart joints produced via conventional FSJ, regardless of the polymeric base material.

The results demonstrate that polymer rheology plays a critical role in influencing material flow, defect formation, and mechanical performance during solid-state joining. These findings provide insights into the relationship between rheological behavior and process stability, highlighting the need to tailor processing parameters so that the flow characteristics of individual polymers enable optimal joint quality and structural integrity.

## Figures and Tables

**Table 1:** Set of parameters employed to produce metal–polymer composite joints.

ω (rpm)	v (mm/min)	α (°)	DT (s)	PL (mm)	PD (mm)
1000	100	2	2.5	1.5	0.9

ω: rotational speed; v: travel speed; α: tilt angle; DT: dwell time; PL; pin length; PD: plunge depth.

**Table 2:** Main physical, thermal, and mechanical properties of Noryl [22], PEEK [3], AA6082-T6 [22] and Ti Gr1 [3].

Property	Noryl GFN2	SustaPEEK	AA6082-T6	Ti Gr.1
Density (g/cm^3^)	1.25	1.31	2.70	4.51
Thermal Conductivity (W/(m°C))	0.26	0.25	180	16
CTE (µm/m°C)	28.9	50	23	9.2
Tg (°C)	143	143	-	-
Tmelt (°C)	280	343	555	1670
Young Modulus (GPa)	6	4	70	105
Tensile Strength (MPa)	80	>110	>290	>240
Fracture Strain (%)	2.5	20	>7.5	24

CTE: coefficient of thermal expansion; Tg: glass transition temperature; Tmelt: melting temperature.

**Table 3:** Tensile testing summary for this study and results available in the literature.

Reference	Base Materials	Thickness	Joining Process	N_UTL_ (kN/m) *
Correia et al. [5]	AA6082-T6 Noryl GFN2	2 mm 5 mm	FSJ	137
Khadka et al. [3]	AA5754-H111 PEEK	5 mm 5 mm	TS-FSJ	110
Ji et al. [35]	AA2024-T4 GFR ** PEEK	4 mm 3 mm	FSJ	135
Huang et al. [36]	AA6061-T6 PEEK	2.5 mm 7 mm	FSJ	100
This study	AA6082-T6 Noryl GFN2	2 mm 5 mm	FSJ	111
This study	AA6082-T6 PEEK	2 mm 5 mm	FSJ	117
This study	AA6082-T6 Noryl GFN2	2 mm 5 mm	TS-FSJ	52
This study	AA6082-T6 PEEK	2 mm 5 mm	TS-FSJ	28

* Determined based on the best joining conditions in this study; ** GFR—glass-fiber-reinforced.

**Table 4:** Comparison of conventional FSJ and TS-FSJ.

	FSJ	TS-FSJ
Process	Friction stir joining	Through-slot friction stir joining
Additional Materials	No additional materials	Uses thin Ti extrusion strips into slots in polymeric material
Joining Process	Stir the metallic material forging it into the polymeric material	Extrude the metallic material through a slot formed by Ti strips into the polymeric material
Heat Input	Generated by friction between tool and metallic material, flowing into the polymeric one by conduction omnidirectionally	Generated by friction between tool and metallic material, localized flow into the polymeric one due to shielding effect imposed by the Ti strips
Mechanical Performance	Higher NUTL; stable for Noryl, scattered for PEEK	Significantly lower NUTL for both polymers
Process Stability	Sensitive to polymer rheology	Sensitive to Ti deformation and heat shielding

## Data Availability

The original contributions presented in this study are included in the article. Further inquiries can be directed to the corresponding author.
